# Association of dietary fibre intake with subsequent fasting glucose levels and indicators of adiposity in school-age Japanese children

**DOI:** 10.1017/S136898002300099X

**Published:** 2023-08

**Authors:** Keiko Wada, Chisato Nagata, Michiyo Yamakawa, Yuma Nakashima, Sachi Koda, Takahiro Uji, Michiko Tsuji, Hideshi Nagai, Naoko Itakura, Kou Harada, Osamu Takahara, Hiromichi Yamanaka

**Affiliations:** 1 Department of Epidemiology and Preventive Medicine, Gifu University Graduate School of Medicine, Gifu 501-1194, Japan; 2 Department of Food Science and Nutrition, Nagoya Women’s University, Nagoya, Japan; 3 Hekinan Medical Association, Hekinan, Japan

**Keywords:** Dietary fibre, Glucose, Weight, School-age children, Epidemiology

## Abstract

**Objective:**

To evaluate the relationships of fibre intake with subsequent BMI sd-score, waist-to-height ratio and serum fasting glucose levels among school-age Japanese children.

**Design:**

This is a prospective study of school-age Japanese children. Participants were followed from 6–7 to 9–10 years of age (follow-up rate: 92·0 %). Fibre intake was assessed using a validated FFQ. Serum fasting glucose was measured by a hexokinase enzymatic method. Using a general linear model, the associations between dietary fibre intake at baseline and BMI sd-score, waist-to-height ratio, and serum levels of fasting glucose at follow-up were evaluated after considering potential confounding factors.

**Setting:**

Public elementary schools in a city in Japan

**Participants:**

A total of 2784 students.

**Results:**

The estimated means for fasting glucose at 9–10 years of age were 86·45, 85·68, 85·88 and 85·58 mg/dl in the lowest, second, third and highest quartile of fibre intake at 6–7 years of age, respectively (*P* = 0·033, trend *P* = 0·018). Higher fibre intake at 6–7 years of age was associated with lower waist-to-height ratio at 9–10 years of age (trend *P* = 0·023). The change in fibre intake was inversely associated with concurrent change of BMI sd-score (trend *P* = 0·044).

**Conclusion:**

These results suggest that dietary fibre intake may be potentially effective to limit excess weight gain and lower glucose levels during childhood.

Obesity and glucose abnormalities in childhood are associated with increased risks of metabolic syndrome and type 2 diabetes in adulthood^(1,2)^. In Japan, the number of adults suspected of having obesity and diabetes has been increasing for several decades and has become an important public health issue^(3)^. Many lifestyle behaviours adopted in childhood and adolescence have been reported to track into adulthood^(4)^. Therefore, it is important to assess the role of lifestyles on childhood adiposity and glucose metabolism.

Dietary fibre consists of non-digestive forms of carbohydrate found in plant foods. It has been suggested that dietary fibre could facilitate body weight and glycemic control through several mechanisms. High-fibre diets might reduce appetite and lead to lower energy intake via prolonged secretion of glucagon-like peptides in the distal small intestine^(5)^. Soluble fibres delay small bowel absorption, which decreases the digestion and absorption of nutrients^(6)^. Insoluble fibres can increase faecal bulk and shorten transit time^(6)^. In addition, increasing insoluble fibre intake also might increase gas and SCFA production, causing longer-lasting satiety and slowing down the acute insulin response^(5,7)^.

Dietary fibre intake has been decreased paralleling with increase in obesity^(8)^. Several prospective cohort studies have indicated associations of wholegrain and fibre intakes with less weight gain and lower risk of diabetes over time^(9–13)^. Randomised controlled trials showed significant reduction in body weight or fasting glucose after dietary wholegrain, pulse or fibre intervention diet compared with comparator diets, although the magnitude of associations was modest^(13–15)^. However, evidence for an association between fibre intake and body weight^(16–22)^ or glucose metabolism^(16,18,22–24)^ in childhood is limited, with conflicting results. Besides, since low intake of dietary fibre among children and adolescents may be of public health concern worldwide^(25–27)^, it is worthwhile to elucidate the health effects of dietary fibre in children.

Thus, the aim of this study was to evaluate the relationships between fibre intake and subsequent BMI, waist circumference and fasting glucose in a prospective study of school-aged children in Japan. We also assessed the association between change in fibre and concurrent change in BMI during follow-up.

## Methods

### Participants and design

**Fig. 1:**
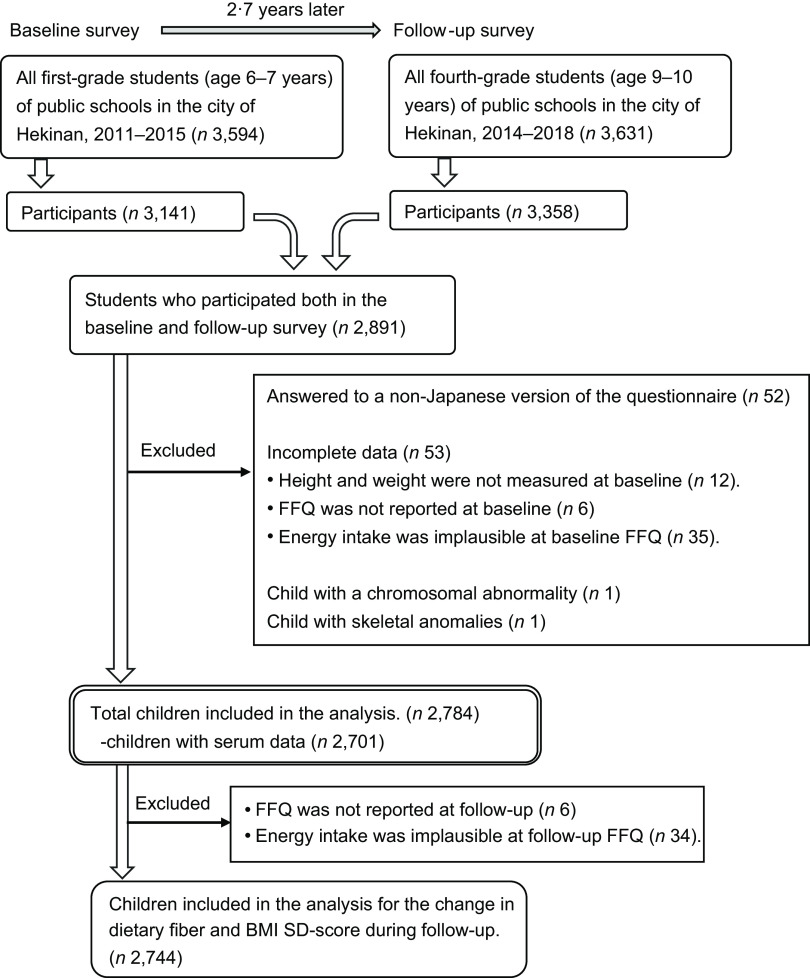
Flow chart of study participants of Hekinan Children’s Study, Japan

Hekinan Children’s Study, a prospective study in Japan, has been previously described elsewhere^(28,29)^. The baseline survey was conducted each autumn from 2011 to 2015. All first-grade elementary students in public schools were invited in the city of Hekinan, Aichi Prefecture. Because there were no private schools there, the study covered almost all residents aged 6–7 years. Of 3594 students, 3141 participated in the baseline survey (participation rate: 87·4 %) (Fig. 1). The baseline survey included a parent-administered questionnaire, a FFQ and anthropometric measurements.

A follow-up survey was performed 3 years later in spring when the participants reached the fourth grade of elementary school. The participants included all fourth-grade students (aged 9–10 years) in the city at that time, whether or not they had participated in the baseline survey. The follow-up survey included parent- and self-administered questionnaires, the FFQ and a health examination. The health examination included waist circumference measurements and the collection of fasting blood. The participation rate was 92·5 % (3358 participants among all 3631 students). Of the 3141 participants in the baseline survey, 2891 participated in the follow-up survey (follow-up rate: 92·0 %).

### Dietary data

Dietary intake was assessed using a validated FFQ^(28,30)^. In the FFQ, the parents were asked how often their children consumed each of the food items listed during the past 6 months and what the usual portion size of each item was. The food list consisted of 162 items for the first-grade children and further added coffee for the fourth-grade children. Nutrient intake was estimated from the frequency of ingestion and portion size using the Japanese Standard Table of Food Composition (5th revised and enlarged edition), published by the Science and Technology Agency of Japan^(31)^. Dietary fibre was defined as total dietary fibre including soluble and insoluble fibre. When compared with two 3-d diet records, the Spearman correlation coefficients for total energy, carbohydrates and dietary fibre were 0·34, 0·38 and 0·37 for the baseline FFQ and 0·28, 0·25 and 0·27 for the follow-up FFQ, respectively^(28,30)^. To assess under-reporting and over-reporting, the dietary data were examined for plausibility of total energy intake. Based on the distribution of the residuals of the linear regression of total energy intake by body weight^(32)^, the dietary data that a residual was > 3·0 sd from the mean were considered invalid.

### Anthropometric measurements and pubertal status

Height and weight were measured at school at the first and the fourth grade. As a rule, height and weight were taken with the participants dressed in light indoor clothing after removing their shoes. Height was measured to the nearest 0·1 cm. Weight was measured to the nearest 0·1 kg. BMI was calculated as weight in kilograms divided by height in metres squared. The sex- and age-independent sd scores for BMI were calculated using the LMS (lambda-mu-sigma) method with Japanese references^(33)^. Waist circumference was measured at the fourth grade. It was taken using a tape measure at the level of umbilicus after expiration to the nearest 0·1 cm while the participant was in the standing position. The waist-to-height ratio was calculated by dividing waist circumference by height.

Adapted from the measure of pubertal status by Petersen et al.^(34)^, several characteristics of pubertal development, such as facial hair growth and voice-deepening change for boys and menarche for girls, were obtained from parent-administered questionnaires at the fourth grade.

### Blood examinations

At the follow-up survey, blood collection was performed immediately after the students arrived at school in the morning. The participants were asked not to eat or drink anything other than water from the night before the blood test. Blood samples were transported to a clinical examination centre in Hekinan after collection. Serum fasting glucose was measured by a hexokinase enzymatic method. Measurements were performed using an automated biochemical analyser (Hitachi 7700; Hitachi High-Tech Science Corp.).

### Potential confounders

The questionnaire at baseline covered birth weight, number of siblings, feeding during infancy, and both parent’s ages, heights, weights, and medical histories. The smoking habits of the parents and other cohabiters were also recorded. Using the timing of smoking initiation and cessation of the parents and cohabiters and the child’s birthday, we calculated the pack-years of household smoking to which the child had been exposed from birth. Physical activity was assessed by the questions adapted from the questionnaire by Booth et al.^(35)^; we asked parents whether their child participated in organised sports, games and other activities supervised by adults. For each physical activity in which the child was involved, the average number of times per week and the average duration of participation were inquired. Time spent in moderate-to-vigorous physical activity (≥ 3·0 metabolic equivalents) was calculated for each child. Time spent in screen viewing was defined as the time children spent in front of a television, video game device and computer. Screen time was inquired separately for weekdays and weekends, so the average time was calculated by adding five-sevenths of the weekday time and two-sevenths of the weekend time. Mother’s and Father’s overweight were defined as BMI of ≥ 25 kg/m^2^.

### Statistical analysis

For the analysis, we excluded those who answered to a non-Japanese version of the questionnaire that did not include FFQ (*n* 52) and had incomplete data (*n* 53) (Fig. 1). Children who had a chromosomal abnormality (*n* 1) or skeletal anomalies (*n* 1) were also excluded because of their potential effect on growth. Finally, a total of 2784 children were included in the analysis. Among them, 2701 measured serum fasting glucose levels.

The participants’ characteristics and nutritional and physical data were described as means and standard deviation or percentages. The intakes of nutrients were controlled for total energy by using the residual method proposed by Willett^(36)^. Participants were divided into quartile groups (Q1, Q2, Q3 and Q4) according to energy-adjusted fibre intake. Since the analyses indicated no interaction by sex for the relationship of fibre intake to body composition and fasting glucose, all analyses were performed with the boys and girls combined.

The estimated means and standard errors for BMI sd-score, waist-to-height ratio and fasting glucose levels were calculated according to the quartiles of fibre intake by using a general linear model. First, we assessed the cross-sectional association between fibre intake and BMI sd-score at baseline. Considering factors that influence child growth or obesity through a literature review, the analyses were adjusted for the following potential confounders: sex (boy, girl), year of entry (2011, 2012, 2013, 2014 and 2015), school (seven schools) and age (continuous, years) at the first grade, and additionally, sibling (yes, no), birth weight (quartiles), feeding at 4 months old (breast-fed, mix-fed and bottle-fed), exposure to household smoking (0, ≤ 6 and > 6 pack-years), mother’s age (< 30, 30–< 35, 35–< 40 and ≥ 40 years), mother’s and father’s overweight (yes and no), physical activity time (tertiles), screen time (quartiles), and intakes of total energy and carbohydrates (quartiles) at the first grade. Next, the associations of fibre intake at baseline with BMI sd-score, waist-to-height ratio and fasting glucose at follow-up were evaluated. In addition, the association with the change in BMI sd-score during follow-up was assessed. These analyses were performed after adjustment for sex, year of entry, and school at the first grade, and age and puberty (beginning both facial hair and voice change for boys and having menarche occurred for girls) (yes, no) at the fourth grade. The models were further adjusted for sibling, birth weight, feeding at 4 months old, exposure to household smoking, mother’s age, mother’s and father’s overweight, physical activity time, screen time, intakes of total energy and carbohydrates, and BMI sd-score (quartiles) at the first grade. Mother’s and father’s history of diabetes were additionally adjusted for in the analysis on fasting glucose. Last, the association between the change in fibre intake and concurrent change in BMI sd-score during follow-up was studied among 2744 children who had both nutritional data at the first and fourth grade (Fig. 1). The covariates were the same as in the analysis on BMI sd-score at follow-up, except that the baseline values were substituted for the change during follow-up for the intakes of total energy and carbohydrates.

Indicator terms were created specifically for missing categorical covariate data. The tests for trend were conducted using the median values of fibre intake by the quartile groups. SAS version 9.4 (SAS Institute) was used for data analysis. All *P*-values were calculated using a two-sided test. A *P*-value less than 0·05 was considered to be statistically significant.

## Results

The participants comprised 1475 boys and 1309 girls. No participant had a history of diabetes, hypertension or lipid metabolism disorder. Overweight was observed in 9·0 % of their mothers and 27·1 % of their fathers (Table 1). The means of child BMI were 15·3 at the first grade and 16·5 at the fourth grade (Table 2). A total of 5·1 % and 0·9 % of participants at the first grade and 6·6 % and 0·4 % at the fourth grade, respectively, were overweight and obesity according to the BMI cut-off points by the International Obesity Task Force (the age- and sex-specific cut-offs that are correspondent to the adult BMI of ≥ 25 for overweight and ≥ 30 for obesity)^(37)^. The mean intakes of total fibre were 11·2 g and 13·6 g per d at the first and fourth grade, respectively.

**Table 1:** Characteristics of 2784 study participants at baseline

	Valid response (*n*)	Percentage	Mean	sd
Age (years)	2784		7·0	0·3
Sex (boys: %)	2784	53·0		
Sibling (yes: %)	2779	90·3		
Birth weight (g)	2721		2982	437
Feeding at 4 months old (%)	2558			
Breast-fed	1438	56·2		
Mix-fed	687	26·9		
Bottle-fed	433	16·9		
Time spent in physical activity ≥ 3·0 METs (h/week)	2738		1·69	2·53
Time spent in screen viewing (h/d)	2685		2·95	1·51
Exposure to household smoking (%)	2383			
None	977	41·0		
<= 6 pack-years	766	32·1		
> 6 pack-years	640	26·9		
Mother’s age (years)	2748		36·5	4·6
Father’s age (years)	2641		38·6	5·4
Mother’s overweight (yes: %)	2600	9·0		
Father’s overweight (yes: %)	2477	27·1		
Mother’s history of diabetes (yes: %)	2784	0·6		
Father’s history of diabetes (yes: %)	2784	1·9		

Values are mean (sd) or percentage.

METs: metabolic equivalents.

**Table 2:** Physical and nutritional data at baseline and at follow-up of 2784 study participants

	1st grade (baseline)			4th grade (follow-up)		
	%	Mean	sd	%	Mean	sd
Age (years)		7·0	0·3		9·6	0·3
Height (cm)		118·0	4·9		132·6	5·8
Weight (kg)		21·4	3·5		29·1	5·5
BMI (kg/m^2^)		15·3	1·7		16·5	2·2
BMI sd-score		−0·36	0·97		−0·26	0·94
BMI classification (%)*						
Thinness	21·6			17·9		
Normal	72·4			75·1		
Overweight	5·1			6·6		
Obesity	0·9			0·4		
Waist circumference (cm)		–			56·6	6·5
Waist-to-height ratio		–			0·43	0·04
Serum fasting glucose†		–			85·9	5·8
Puberty (yes: %)‡		–		0·3		
Nutritional data§						
Total energy intake (kcal/d)		1621	418		2078	521
Carbohydrate intake (g/d)		219·6	57·5		281·7	72·4
Fibre intake (g/d)		11·2	3·7		13·6	4·1
Fibre intake (g/1000 kcal)		6·9	1·3		6·6	1·2

Values are mean (sd) or percentage.

* BMI was classified according to the cut-offs by the International Obesity Task Force: age- and sex-specific cut-off points corresponding to the adult BMI of < 18·5 for thinness, ≥ 25 for overweight and ≥ 30 for obesity, respectively.

† Serum fasting glucose was obtained for 2701 children.

‡ Puberty was defined as beginning of both facial hair and voice change for boys and experiencing menarche for girls.

§ Nutritional data at follow-up were obtained for 2744 children.

We observed a significant cross-sectional association between higher intake of dietary fibre and lower BMI sd-score at the first grade (trend *P* = 0·036) (Table 3). A higher intake of dietary fibre at the first grade was associated with lower waist-to-height ratio at the fourth grade (trend *P* = 0·023). Fibre intake at the first grade was inversely associated with fasting glucose at the fourth grade (*P* = 0·033, trend *P* = 0·018). The change in fibre intake was inversely associated with concurrent change of BMI sd-score during follow-up (trend *P* = 0·044) (Table 4).

**Table 3:** Associations of fibre intake at the 1st grade with BMI sd-score, waist-to-height ratio and serum fasting glucose during follow-up among 2784 children

	Fibre intake at the 1st grade (median: g/d)											
	Q1 (9·0)		Q2 (10·4)		Q3 (11·5)		Q4 (13·5)			Trend		
	Mean	se	Mean	se	Mean	se	Mean	se	*P*	*β*	se	*P*
*n*	696		696		696		696					
BMI sd-score at the 1st grade												
Model 1	−0·242	0·037	−0·388	0·036	−0·392	0·036	−0·408	0·037	0·004	−0·033	0·011	0·004
Model 2	−0·307	0·035	−0·350	0·035	−0·358	0·035	−0·414	0·035	0·21	−0·023	0·011	0·036
BMI sd-score at the 4th grade												
Model 3	−0·132	0·036	−0·293	0·035	−0·298	0·036	−0·313	0·036	<0·001	−0·036	0·011	0·001
Model 4	−0·226	0·020	−0·267	0·020	−0·259	0·020	−0·283	0·020	0·24	−0·011	0·006	0·071
Change in BMI sd-score from the 1st grade to the 4th grade												
Model 3	0·111	0·017	0·096	0·016	0·097	0·016	0·091	0·017	0·85	−0·004	0·005	0·43
Model 4	0·113	0·016	0·087	0·016	0·097	0·016	0·097	0·016	0·74	−0·002	0·005	0·62
Waist-to-height ratio at the 4th grade												
Model 3	0·432	0·002	0·426	0·002	0·424	0·002	0·423	0·002	<0·001	−0·002	0·000	<0·001
Model 4	0·429	0·001	0·427	0·001	0·426	0·001	0·425	0·001	0·14	−0·001	0·000	0·023
Fasting glucose at the 4th grade*												
Model 3	86·60	0·22	85·69	0·22	85·83	0·22	85·46	0·22	0·002	−0·221	0·068	0·001
Model 5	86·45	0·23	85·68	0·22	85·88	0·22	85·58	0·22	0·033	−0·163	0·069	0·018

Values are estimated mean (se). Dietary fibre intakes were controlled for total energy using the residual method.

Model 1 was adjusted for sex (boy, girl), year of entry (2011, 2012, 2013, 2014, 2015), school (7 schools) and age (years) at the 1st grade.

Model 2 was adjusted for sibling (yes, no), birth weight (quartiles), feeding at 4 months old (breast-fed, mix-fed, bottle-fed), exposure to household smoking (0, ≤ 6, > 6 pack-years), mother’s age (< 30, 30–< 35, 35–< 40, ≥ 40 years), mother’s and father’s overweight (yes, no), physical activity time (tertiles), screen time (quartiles), and intake of total energy and carbohydrates (quartiles) at the 1st grade, in addition to the variables included in model 1.

Model 3 was adjusted for sex (boy and girl), year of entry (2011, 2012, 2013, 2014 and 2015) and school (seven schools) at the 1st grade and age (years) and puberty (yes and no) at the 4th grade.

Model 4 was adjusted for sibling (yes and no), birth weight (quartiles), feeding at 4 months old (breast-fed, mix-fed and bottle-fed), exposure to household smoking (0, ≤ 6 and > 6 pack-years), mother’s age (< 30, 30–< 35, 35–< 40 and ≥ 40 years), mother’s and father’s overweight (yes and no), physical activity time (tertiles), screen time (quartiles), intake of total energy and carbohydrates (quartiles) and BMI sd-score (quartiles) at the 1st grade, in addition to the variables included in model 3.

Model 5 was adjusted for sibling (yes and no), birth weight (quartiles), feeding at 4 months old (breast-fed, mix-fed and bottle-fed), exposure to household smoking (0, ≤ 6 and > 6 pack-years), mother’s age (< 30, 30–< 35, 35–< 40 and ≥ 40 years), mother’s and father’s overweight (yes and no), mother’s and father’s history of diabetes (yes and no), physical activity time (tertiles), screen time (quartiles), intake of total energy and carbohydrates (quartiles) and BMI sd-score (quartiles) at the 1st grade, in addition to the variables included in model 3.

* The analyses on serum glucose are performed among 2701 participants.

**Table 4:** Association between change in fibre intake and concurrent change in BMI sd-score from the 1st grade to the 4th grade among 2744 children

	Change in fibre intake (median: g/d)											
	Q1 (0·1)		Q2 (1·8)		Q3 (2·9)		Q4 (4·7)			Trend		
	Mean	se	Mean	se	Mean	se	Mean	se	*P*	*β*	se	*P*
*n*	686		686		686		686					
Change in BMI sd-score												
Model 1	0·128	0·017	0·103	0·017	0·084	0·017	0·077	0·017	0·13	−0·011	0·005	0·022
Model 2	0·124	0·016	0·105	0·016	0·083	0·016	0·079	0·016	0·18	−0·010	0·005	0·044

Values are estimated mean (se). Dietary fibre intakes were controlled for total energy using the residual method.

Model 1 was adjusted for sex (boy and girl), year of entry (2011, 2012, 2013, 2014 and 2015) and school (seven schools) at the 1st grade and age (years) and puberty (yes and no) at the 4th grade.

Model 2 was adjusted for sibling (yes and no), birth weight (quartiles), feeding at 4 months old (breast-fed, mix-fed and bottle-fed), exposure to household smoking (0, ≤ 6 and > 6 pack-years), mother’s age (< 30, 30–< 35, 35–< 40 and ≥ 40 years), mother’s and father’s overweight (yes and no), physical activity time (tertiles), screen time (quartiles), BMI sd-score (quartiles) at the 1st grade, and the change in the intake of total energy and carbohydrates (quartiles), in addition to the variables included in model 1.

For sensitivity analysis, we additionally adjusted for fat intake. The significant associations of higher fibre intakes at the first grade with waist-to-height ratio (trend *P* = 0·047) and lower fasting glucose (trend *P* = 0·040) at the fourth grade remained. The change in fibre intake was inversely associated with concurrent change of BMI sd-score (trend *P* = 0·046).

When we reanalysed the associations after excluding one boy whose both facial hair and voice change began and eight girls who experienced menarche, no substantial changes were observed; the inverse association between fibre intakes at the first grade and fasting glucose at the fourth grade was significant (*P* = 0·035, trend *P* = 0·019). Higher fibre intakes at the first grade were associated with lower waist-to-height ratio at the fourth grade (trend *P* = 0·017). The change in fibre intake was inversely associated with the change in BMI sd-score (trend *P* = 0·041).

Fibre intake originated from vegetables (41 %), cereals (19 %), fruits (7 %), pulses (6 %), potatoes and starches (5 %), mushrooms (5 %), algae (5 %) and others among first-grade children, and the composition of fibre food source did not largely change at the fourth grade. When we analysed for the intakes of vegetable fibre, cereal fibre and fruit fibre, there was a significant inverse association between vegetable fibre intake at the first grade and fasting glucose at the fourth grade (trend *P* = 0·008). Fruit fibre intake was inversely associated with waist-to-height ratio (trend *P* < 0·001) (online Supplementary Table 1 and 2). When divided into soluble and insoluble fibre, both soluble and insoluble fibre were associated with waist-to-height ratio (both trend *P* = 0·004), but only insoluble fibre was associated with fasting glucose (trend *P* = 0·022).

Last, we repeated the analyses without adjusting dietary fibre for total energy intake (online Supplementary Table 3 and 4). The significant associations of higher fibre intakes at the first grade with lower waist-to-height ratio (trend *P* = 0·039) and fasting glucose (trend *P* = 0·049) at the fourth grade were not altered.

## Discussion

This study demonstrated that higher intakes of dietary fibre intake at 6–7 years of age was associated with lower fasting glucose level and waist-to-height ratio at 9–10 years of age in Japanese children. In addition, the 2·7-year change in fibre intake was inversely associated with concurrent change in BMI sd-score.

Only a few studies have assessed the longitudinal association between dietary fibre intake and glucose–insulin metabolism in children, in which the results were inconsistent^(16,18,22–24)^. Fibre intake was positively associated with insulin secretion 2 years later among children aged 8–10 years in Canada^(23)^. An increase in soluble fibre intake between the ages of 16–17 years and 18–19 years was associated with concurrently improved insulin sensitivity among girls in the UK^(24)^. In a 16-week intervention study of nutrition and exercise programmes (mean age: 15 years), overweight Latino children who increased their fibre intake showed improvements in insulin sensitivity^(16)^. However, there were no associations of fibre intake with glucose and insulin indices among Danish children^(22)^ and overweight Latino children^(18)^. These inconsistent findings might partly be explained by differences in participant characteristics, the dietary assessment methods used and potential confounding factors considered.

Several epidemiological studies have reported that increased fibre intake is longitudinally associated with decreased BMI^(16,17)^, visceral adiposity^(16,18)^, body fat percentage^(19)^ and waist circumference^(17)^ in childhood, although some other studies found no such associations^(20–22)^. In the 16-week randomised controlled trial described above, participants who increased their fibre intake had a significant decrease in BMI compared with those who did not^(16)^. Among Australian students, the change in fibre intake over 5 years from the age of 12 years was associated with a concurrent decrease in waist circumference in boys and a decrease in BMI in girls^(17)^. Although these magnitudes of the association were modest similar to this study, such minor improvement of weight and adiposity by fibre intake in childhood may be important for long-term obesity risk later in life^(1,2)^.

Fibre food source might be responsible for the effects of dietary fibre on weight change and glucose metabolism. In this study, fruit and vegetable fibre seemed to play a greater role than cereal fibre in relation to waist-to-height ratio and fasting glucose. Besides, the findings related to fibre intake may reflect the effects of a healthy diet containing fibre (low-calorie and low-fat diet) rather than fibre consumption *per se*. However, when we additionally adjusted for fat intake, the associations observed were not altered substantially.

The FFQ was designed to measure an individual’s relative intakes of foods and nutrients rather than absolute values, and the values for fibre intake may have been overestimated in our FFQ^(30)^. However, many students’ fibre intakes in this study were lower compared with the sex- and age-based Japanese dietary reference intake values (the tentative dietary goal: 5·8–6·5 g/1000 kcal for boys and 5·9–6·9 g/1000 kcal for girls aged 6–11 years)^(38)^ and the recommended amount according to the dietary reference intake for American children aged 2 years and older (14 g/1000 kcal)^(39)^. Fibre intakes by dietary records^(16,19,21,22)^, diet recall^(18)^ and FFQ^(17,20)^ in previous studies of children on dietary fibre and body composition were also less than the recommendation. Relatively higher levels of fibre intake can provide more significant change on child’s weight and glucose metabolism.

The key strength of this study includes its longitudinal design, relatively large sample size and use of a validated FFQ to measure habitual dietary intake. Dietary information was collected twice, allowing us to explore the concurrent associations between changes in fibre intake and BMI over time. The high participation rate, high follow-up rate and adjustment for various potential confounders including lifestyle and familial factors were also advantages. Nevertheless, some potential limitations need to be addressed. First, waist circumference measurement and blood collection were performed at follow-up only, and so it was not possible to evaluate those changes during follow-up. Next, since the correlation coefficients between FFQ and the diet records were not so high, some degree of measurement error in fibre intake might have occurred. However, since it is well known that overweight/obese people are prone to over-reporting consumption of healthy foods and under-reporting of energy, the fibre intake of children with higher BMI would have tended to be over-reported by their parents. Such misclassification might have led to an underestimation of the associations observed. In addition, the validity of our FFQ was similar to that of other nutritional epidemiological studies of children^(30)^. Lastly, the children studied in this study were somewhat slimmer than the Japanese reference population with the same age (the mean BMI sd-score: –0·36), which may affect the generalisability of our findings. Meanwhile, the average fibre intake in this study was similar to that in two other school-based studies in Japan^(26,40)^.

In conclusion, this study suggested that dietary fibre intake at the age of 6–7 years was inversely associated with serum levels of fasting glucose and waist-to-height ratio at the age of 9–10 years in Japanese children. The 2·7-year change in fibre intake from 6–7 to 9–10 years of age was inversely associated with concurrent change in BMI sd-score. Dietary fibre intake may be potentially effective to limit excess weight gain and lower glucose levels during childhood.

## Supplementary material

For supplementary material accompanying this paper visit https://doi.org/10.1017/S136898002300099X. click here to view supplementary material
