# Supplementary material for: Association of dietary fibre intake with subsequent fasting glucose levels and indicators of adiposity in school-age Japanese children
Source: Public Health Nutr. 2023 May 25;26(8):1617–25. doi: 10.1017/S136898002300099X (PMC10410373; doi:10.1017/S136898002300099X)
Supplement: Supplementary file 1 [file S136898002300099Xsup001.docx]

| **Supplementary table 1** Associations of intake of various types of fiber at the 1st grade with BMI SD-score, waist-to-height ratio, and serum fasting glucose during follow-up among 2,784 children. | | | | | | | | | | | | | |
| --- | --- | --- | --- | --- | --- | --- | --- | --- | --- | --- | --- | --- | --- |
|  | Fiber intake in the 1st grade | | | |  |  |  |  |  |  |  |  |  |
|  | Q1 |  |  | Q2 | |  | Q3 |  |  | Q4 |  | *p* | *trend p* |
|  | mean | (SE) |  | mean | (SE) |  | mean | (SE) |  | mean | (SE) |  |  |
| *n* | 696 |  |  | 696 |  |  | 696 |  |  | 696 |  |  |  |
| Cereal fiber |  |  |  |  |  |  |  |  |  |  |  |  |  |
| BMI SD-score at the 1st grade^a^ | -0.411 | (0.036) |  | -0.334 | (0.035) |  | -0.348 | (0.035) |  | -0.337 | (0.035) | 0.38 | 0.20 |
| BMI SD-score at the 4th grade^b^ | -0.241 | (0.020) |  | -0.275 | (0.020) |  | -0.256 | (0.020) |  | -0.263 | (0.020) | 0.69 | 0.59 |
| Change in BMI SD-score from the 1st grade to the 4th grade^b^ | 0.102 | (0.016) |  | 0.083 | (0.016) |  | 0.110 | (0.016) |  | 0.098 | (0.016) | 0.67 | 0.90 |
| Waist-to-height ratio at the 4th grade^b^ | 0.427 | (0.001) |  | 0.426 | (0.001) |  | 0.427 | (0.001) |  | 0.426 | (0.001) | 0.78 | 0.64 |
| Fasting glucose at the 4th grade^c,d^ | 86.08 | (0.23) |  | 85.72 | (0.22) |  | 85.94 | (0.22) |  | 85.83 | (0.22) | 0.70 | 0.57 |
| Vegetable fiber |  |  |  |  |  |  |  |  |  |  |  |  |  |
| BMI SD-score at the 1st grade^a^ | -0.321 | (0.036) |  | -0.347 | (0.035) |  | -0.364 | (0.035) |  | -0.398 | (0.035) | 0.49 | 0.12 |
| BMI SD-score at the 4th grade^b^ | -0.229 | (0.020) |  | -0.282 | (0.020) |  | -0.253 | (0.020) |  | -0.272 | (0.020) | 0.25 | 0.27 |
| Change in BMI SD-score from the 1st grade to the 4th grade^b^ | 0.112 | (0.016) |  | 0.066 | (0.016) |  | 0.116 | (0.016) |  | 0.099 | (0.016) | 0.11 | 0.94 |
| Waist-to-height ratio at the 4th grade^b^ | 0.429 | (0.001) |  | 0.425 | (0.001) |  | 0.427 | (0.001) |  | 0.425 | (0.001) | 0.064 | 0.058 |
| Fasting glucose at the 4th grade^c,d^ | 86.39 | (0.23) |  | 85.70 | (0.22) |  | 86.07 | (0.22) |  | 85.42 | (0.22) | 0.013 | 0.008 |
| Fruit fiber |  |  |  |  |  |  |  |  |  |  |  |  |  |
| BMI SD-score at the 1st grade^a^ | -0.333 | (0.036) |  | -0.329 | (0.035) |  | -0.355 | (0.035) |  | -0.412 | (0.036) | 0.33 | 0.076 |
| BMI SD-score at the 4th grade^b^ | -0.234 | (0.021) |  | -0.253 | (0.020) |  | -0.246 | (0.020) |  | -0.302 | (0.020) | 0.096 | 0.018 |
| Change in BMI SD-score from the 1st grade to the 4th grade^b^ | 0.110 | (0.017) |  | 0.096 | (0.016) |  | 0.098 | (0.016) |  | 0.090 | (0.016) | 0.86 | 0.46 |
| Waist-to-height ratio at the 4th grade^b^ | 0.430 | (0.001) |  | 0.427 | (0.001) |  | 0.426 | (0.001) |  | 0.423 | (0.001) | 0.004 | <0.001 |
| Fasting glucose at the 4th grade^c,d^ | 85.80 | (0.23) |  | 86.11 | (0.22) |  | 86.05 | (0.22) |  | 85.62 | (0.22) | 0.40 | 0.34 |
| Soluble fiber |  |  |  |  |  |  |  |  |  |  |  |  |  |
| BMI SD-score at the 1st grade^a^ | -0.302 | (0.035) |  | -0.357 | (0.035) |  | -0.343 | (0.035) |  | -0.427 | (0.035) | 0.086 | 0.016 |
| BMI SD-score at the 4th grade^b^ | -0.224 | (0.020) |  | -0.258 | (0.020) |  | -0.259 | (0.020) |  | -0.294 | (0.020) | 0.10 | 0.016 |
| Change in BMI SD-score from the 1st grade to the 4th grade^b^ | 0.111 | (0.016) |  | 0.102 | (0.016) |  | 0.085 | (0.016) |  | 0.095 | (0.016) | 0.71 | 0.43 |
| Waist-to-height ratio at the 4th grade^b^ | 0.429 | (0.001) |  | 0.427 | (0.001) |  | 0.426 | (0.001) |  | 0.424 | (0.001) | 0.041 | 0.004 |
| Fasting glucose at the 4th grade^c,d^ | 86.23 | (0.22) |  | 85.83 | (0.22) |  | 85.78 | (0.22) |  | 85.73 | (0.22) | 0.37 | 0.13 |
| Insoluble fiber |  |  |  |  |  |  |  |  |  |  |  |  |  |
| BMI SD-score at the 1st grade^a^ | -0.300 | (0.036) |  | -0.335 | (0.035) |  | -0.371 | (0.035) |  | -0.424 | (0.035) | 0.085 | 0.010 |
| BMI SD-score at the 4th grade^b^ | -0.219 | (0.020) |  | -0.260 | (0.020) |  | -0.268 | (0.020) |  | -0.288 | (0.020) | 0.11 | 0.021 |
| Change in BMI SD-score from the 1st grade to the 4th grade^b^ | 0.117 | (0.016) |  | 0.080 | (0.016) |  | 0.101 | (0.016) |  | 0.095 | (0.016) | 0.46 | 0.55 |
| Waist-to-height ratio at the 4th grade^b^ | 0.429 | (0.001) |  | 0.427 | (0.001) |  | 0.426 | (0.001) |  | 0.424 | (0.001) | 0.035 | 0.004 |
| Fasting glucose at the 4th grade^c,d^ | 86.37 | (0.23) |  | 85.69 | (0.22) |  | 86.01 | (0.22) |  | 85.51 | (0.22) | 0.038 | 0.022 |
| Values are estimated mean (SE). Dietary fiber intakes were controlled for total energy using the residual method. | | | | | |  |  |  |  |  |  |  |  |
| SE: standard error, BMI: body mass index, SD-score: standard deviation score. | |  |  |  |  |  |  |  |  |  |  |  |  |
| ^a^Adjusted for sex (boy, girl), year of entry (2011, 2012, 2013, 2014, 2015), school (7 schools), age (years) at the 1st grade, sibling (yes, no), birth weight (quartiles), feeding at 4 months old (breast-fed, mix-fed, bottle-fed), exposure to household smoking (0, ≤6, >6 pack-years), mother's age (<30, 30–<35, 35–<40, ≥40 years), mother's and father's overweight (yes, no), physical activity time (tertiles), screen time (quartiles), and intake of total energy and carbohydrates (quartiles) at the 1st grade | | | | | | | | | | | | | |
| ^b^Adjusted for sex (boy, girl), year of entry (2011, 2012, 2013, 2014, 2015) and school (7 schools) at the 1st grade and age (years), puberty (yes, no) at the 4th grade, sibling (yes, no), birth weight (quartiles), feeding at 4 months old (breast-fed, mix-fed, bottle-fed), exposure to household smoking (0, ≤6, >6 pack-years), mother's age (<30, 30–<35, 35–<40, ≥40 years), mother's and father's overweight (yes, no), physical activity time (tertiles), screen time (quartiles), intake of total energy and carbohydrates (quartiles) and BMI SD-score (quartiles) at the 1st grade. | | | | | | | | | | | | | |
| ^c^Adjusted for sex (boy, girl), year of entry (2011, 2012, 2013, 2014, 2015) and school (7 schools) at the 1st grade and age (years), puberty (yes, no) at the 4th grade, sibling (yes, no), birth weight (quartiles), feeding at 4 months old (breast-fed, mix-fed, bottle-fed), exposure to household smoking (0, ≤6, >6 pack-years), mother's age (<30, 30–<35, 35–<40, ≥40 years), mother's and father's overweight (yes, no), mother's and father's history of diabetes (yes, no), physical activity time (tertiles), screen time (quartiles), intake of total energy and carbohydrates (quartiles) and BMI SD-score (quartiles) at the 1st grade | | | | | | | | | | | | | |
| ^d^The anlyses on serum glucose are performed among 2,701 participantss. | | | | | | | | | | | | | |

| **Supplementary table 2** Association between change in intake of various types of fiber and concurrent change in BMI SD-score from the 1st grade to the 4th grade among 2,744 children | | | | | | | | | | | | | |
| --- | --- | --- | --- | --- | --- | --- | --- | --- | --- | --- | --- | --- | --- |
|  | Change in fiber intake | | | |  |  |  |  |  |  |  |  |  |
|  | Q1 |  |  | Q2 | |  | Q3 |  |  | Q4 |  | *p* | *trend p* |
|  | mean | (SE) |  | mean | (SE) |  | mean | (SE) |  | mean | (SE) |  |  |
| *n* | 686 |  |  | 686 |  |  | 686 |  |  | 686 |  |  |  |
| Cereal fiber | 0.113 | (0.016) |  | 0.088 | (0.016) |  | 0.101 | (0.016) |  | 0.091 | (0.016) | 0.69 | 0.43 |
| Vegetable fiber | 0.110 | (0.016) |  | 0.084 | (0.016) |  | 0.120 | (0.016) |  | 0.078 | (0.016) | 0.19 | 0.34 |
| Fruit fiber | 0.102 | (0.016) |  | 0.103 | (0.016) |  | 0.088 | (0.016) |  | 0.098 | (0.016) | 0.91 | 0.71 |
| Soluble fiber | 0.120 | (0.016) |  | 0.087 | (0.016) |  | 0.109 | (0.016) |  | 0.075 | (0.016) | 0.19 | 0.12 |
| Insoluble fiber | 0.122 | (0.016) |  | 0.112 | (0.016) |  | 0.072 | (0.016) |  | 0.085 | (0.016) | 0.10 | 0.055 |
| Values are estimated mean (SE). Dietary fiber intakes were controlled for total energy using the residual method. | | | | | | | | | | | | | |
| SE: standard error, BMI: body mass index, SD-score: standard deviation score. | | | | | | | | | | | | | |
| Adjusted for sex (boy, girl), year of entry (2011, 2012, 2013, 2014, 2015) and school (7 schools) at the 1st grade, age (years) and puberty (yes, no) at the 4th grade, sibling (yes, no), birth weight (quartiles), feeding at 4 months old (breast-fed, mix-fed, bottle-fed), exposure to household smoking (0, ≤6, >6 pack-years), mother's age (<30, 30–<35, 35–<40, ≥40 years), mother's and father's overweight (yes, no), physical activity time (tertiles), screen time (quartiles), BMI SD-score (quartiles) at the 1st grade, and the change in the intake of total energy and carbohydrates (quartiles). | | | | | | | | | | | | | |

| **Supplementary table 3** Associations of fiber intake at the 1st grade with BMI SD-score, waist-to-height ratio, and serum fasting glucose during follow-up among 2,784 children. | | | | | | | | | | | | | |
| --- | --- | --- | --- | --- | --- | --- | --- | --- | --- | --- | --- | --- | --- |
|  | Fiber intake at the 1st grade (median: g/day) | | | | | | |  |  |  |  |  |  |
|  | Q1 (7.4) | |  | Q2 (9.5) | |  | Q3 (11.7) | |  | Q4 (15.3) | | *p* | *trend p* |
|  | mean | (SE) |  | mean | (SE) |  | mean | (SE) |  | mean | (SE) |  |  |
| *n* | 696 |  |  | 696 |  |  | 696 |  |  | 696 |  |  |  |
| BMI SD-score at the 1st grade^a^ | -0.297 | (0.045) |  | -0.347 | (0.036) |  | -0.407 | (0.036) |  | -0.379 | (0.044) | 0.37 | 0.31 |
| BMI SD-score at the 4th grade^b^ | -0.190 | (0.025) |  | -0.294 | (0.021) |  | -0.266 | (0.021) |  | -0.286 | (0.025) | 0.010 | 0.11 |
| Change in BMI SD-score from the 1st grade to the 4th grade^b^ | 0.135 | (0.020) |  | 0.069 | (0.017) |  | 0.110 | (0.017) |  | 0.079 | (0.020) | 0.032 | 0.26 |
| Waist-to-height ratio at the 4th grade^b^ | 0.430 | (0.002) |  | 0.425 | (0.001) |  | 0.426 | (0.001) |  | 0.424 | (0.001) | 0.042 | 0.039 |
| Fasting glucose at the 4th grade^c,d^ | 86.47 | (0.28) |  | 86.15 | (0.23) |  | 85.41 | (0.23) |  | 85.55 | (0.28) | 0.046 | 0.049 |
| Values are estimated mean (SE). |  |  |  |  |  |  |  |  |  |  |  |  |  |
| SE: standard error, BMI: body mass index, SD-score: standard deviation score. | | |  |  |  |  |  |  |  |  |  |  |  |
| ^a^Adjusted for sex (boy, girl), year of entry (2011, 2012, 2013, 2014, 2015), school (7 schools), age (years) at the 1st grade, sibling (yes, no), birth weight (quartiles), feeding at 4 months old (breast-fed, mix-fed, bottle-fed), exposure to household smoking (0, ≤6, >6 pack-years), mother's age (<30, 30–<35, 35–<40, ≥40 years), mother's and father's overweight (yes, no), physical activity time (tertiles), screen time (quartiles), and intake of total energy and carbohydrates (quartiles) at the 1st grade | | | | | | | | | | | | | |
| ^b^Adjusted for sex (boy, girl), year of entry (2011, 2012, 2013, 2014, 2015) and school (7 schools) at the 1st grade and age (years), puberty (yes, no) at the 4th grade, sibling (yes, no), birth weight (quartiles), feeding at 4 months old (breast-fed, mix-fed, bottle-fed), exposure to household smoking (0, ≤6, >6 pack-years), mother's age (<30, 30–<35, 35–<40, ≥40 years), mother's and father's overweight (yes, no), physical activity time (tertiles), screen time (quartiles), intake of total energy and carbohydrates (quartiles) and BMI SD-score (quartiles) at the 1st grade. | | | | | | | | | | | | | |
| ^c^Adjusted for sex (boy, girl), year of entry (2011, 2012, 2013, 2014, 2015) and school (7 schools) at the 1st grade and age (years), puberty (yes, no) at the 4th grade, sibling (yes, no), birth weight (quartiles), feeding at 4 months old (breast-fed, mix-fed, bottle-fed), exposure to household smoking (0, ≤6, >6 pack-years), mother's age (<30, 30–<35, 35–<40, ≥40 years), mother's and father's overweight (yes, no), mother's and father's history of diabetes (yes, no), physical activity time (tertiles), screen time (quartiles), intake of total energy and carbohydrates (quartiles) and BMI SD-score (quartiles) at the 1st grade | | | | | | | | | | | | | |
| ^d^The anlyses on serum glucose are performed among 2,701 participants. | | | | | | | | | | | | | |

| **Supplementary table 4** Association between change in fiber intake and concurrent change in BMI SD-score from the 1st grade to the 4th grade among 2,744 children | | | | | | | | | | | | | |
| --- | --- | --- | --- | --- | --- | --- | --- | --- | --- | --- | --- | --- | --- |
|  | Change in fiber intake (median: g/day) | | | | |  |  |  |  |  |  |  |  |
|  | Q1 (-1.1) | |  | Q2 (1.5) | |  | Q3 (3.2) | |  | Q4 (6.2) | | *p* | *trend p* |
|  | mean | (SE) |  | mean | (SE) |  | mean | (SE) |  | mean | (SE) |  |  |
| *n* | 686 |  |  | 686 |  |  | 686 |  |  | 686 |  |  |  |
| Change in BMI SD-score | 0.114 | (0.019) |  | 0.114 | (0.017) |  | 0.077 | (0.017) |  | 0.086 | (0.020) | 0.41 | 0.32 |
| Values are estimated mean (SE). | | | | | | | | | | | | | |
| SE: standard error, BMI: body mass index, SD-score: standard deviation score. | | | | | | | | | | | | | |
| Adjusted for sex (boy, girl), year of entry (2011, 2012, 2013, 2014, 2015) and school (7 schools) at the 1st grade, age (years) and puberty (yes, no) at the 4th grade, sibling (yes, no), birth weight (quartiles), feeding at 4 months old (breast-fed, mix-fed, bottle-fed), exposure to household smoking (0, ≤6, >6 pack-years), mother's age (<30, 30–<35, 35–<40, ≥40 years), mother's and father's overweight (yes, no), physical activity time (tertiles), screen time (quartiles), BMI SD-score (quartiles) at the 1st grade, and the change in the intake of total energy and carbohydrates (quartiles). | | | | | | | | | | | | | |
